# *Mychonastes afer *HSO-3-1 as a potential new source of biodiesel

**DOI:** 10.1186/1754-6834-4-47

**Published:** 2011-11-01

**Authors:** Cheng Yuan, Junhan Liu, Yong Fan, Xiaohui Ren, Guangrong Hu, Fuli Li

**Affiliations:** 1 Shandong Provincial Key Laboratory of Energy Genetics, Qingdao Institute of Bioenergy and Bioprocess Technology, Chinese Academy of Sciences, Qingdao 266101, PR China; 2 Ocean College of Hebei Agriculture University, Qinhuangdao, 066003, PR China

## Abstract

**Background:**

Biodiesel is considered to be a promising future substitute for fossil fuels, and microalgae are one source of biodiesel. The ratios of lipid, carbohydrates and proteins are different in different microalgal species, and finding a good strain for oil production remains a difficult prospect. Strains producing valuable co-products would improve the viability of biofuel production.

**Results:**

In this study, we performed sequence analysis of the 18S rRNA gene and internal transcribed spacer (ITS) of an algal strain designated HSO-3-1, and found that it was closely related to the *Mychonastes afer *strain CCAP 260/6. Morphology and cellular structure observation also supported the identification of strain HSO-3-1 as *M. afer*. We also investigated the effects of nitrogen on the growth and lipid accumulation of the naturally occurring *M. afer *HSO-3-1, and its potential for biodiesel production. In total, 17 fatty acid methyl esters (FAMEs) were identified in *M. afer *HSO-3-1, using gas chromatography/mass spectrometry. The total lipid content of *M. afer *HSO-3-1 was 53.9% of the dry cell weight, and we also detected nervonic acid (C24:1), which has biomedical applications, making up 3.8% of total fatty acids. The highest biomass and lipid yields achieved were 3.29 g/l and 1.62 g/l, respectively, under optimized conditions.

**Conclusion:**

The presence of octadecenoic and hexadecanoic acids as major components, with the presence of a high-value component, nervonic acid, renders *M. afer *HSO-3-1 biomass an economic feedstock for biodiesel production.

## Background

To meet the rising demand for energy resources and the need to protect the environment, renewable biofuels are needed to replace petroleum-derived transport fuels. Biodiesel, from sources such as microalgae, has received extensive attention in recent years, owing to its biodegradability, renewability, and lack of toxicity, among other advantages. However, technical and economic barriers must be overcome to realize the potential of this energy source [1-3].

Despite decades of development, the technology remains in its infancy. For example, problems remain with algal culture methods, inefficient harvesting of and bioenergy extraction from microalgae cells, and poor light penetration in dense microalgal cultures [1,4,5]. In order for microalgae to become an economically viable biofuel feedstock, the cost of producing biodiesel from microalgae needs to be reduced [6]. Identifying a good strain for oil production, which should feature high lipid content, high biomass and tolerance to extreme environments, remains a difficult prospect [6].

It has been suggested that strains producing valuable co-products, such as feed, fertilizers or pharmaceuticals, would further improve the viability of biofuel production [1,7,8]. To date, omega-3 fatty acid, eicosapentanoic acid, decosahexaenoic acid and chlorophyll have been shown to be potentially valuable co-products of microalgal biodiesel production [9-11].

The ratios of lipid, carbohydrates and proteins are different in different microalgal species. In some species, lipids can be up to 60% of the algal dry weight [12,13]. In one exceptional case, a lipid content of 86% dry weight was reported for the brown resting state colonies of *Botryococcus braunii *[14]. Factors such as temperature, irradiation, and most markedly, nutrient availability, have been shown to be crucial to microalgal metabolism, and high lipid productivity can therefore be achieved by optimization of these factors [15,16].

The aims of this work were to discover new microalgae species with high lipid content, yield and suitable fatty acids for biodiesel production. In our previous work, we analyzed the biomass, lipid content and fatty acid (FA) composition of 77 microalgae strains isolated from Shandong province and Beijing, China. Strain HSO-3-1 produced lipids up to 53.9% of its dry weight. Meanwhile, fatty acid analysis of strain HSO-3-1 by GC-MS indicated the presence of C24:1 (nervonic acid, 14.7 mg/g of total lipid, 3.8% of the total fatty acid content), which has been shown to have potential in biomedical applications. So we carried out further investigations on strain HSO-3-1.

## Results and Discussion

### Molecular identification

**Figure 1:**
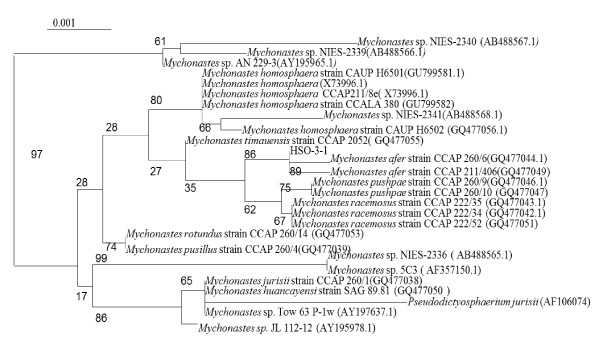
**Phylogenetic tree inferred from 18S rRNA gene sequences**. The distances within the tree were constructed using the neighbor-joining method based on Kimura's correction with the CLUSTAL W computer program. The horizontal lengths are proportional to the evolutionary distances. The numbers above or below the internal nodes indicate bootstrap values (1000 replicates).

**Figure 2:**
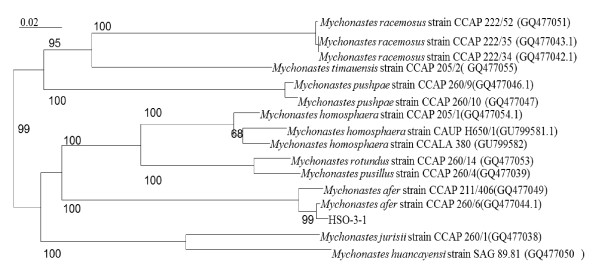
**Phylogenetic tree inferred from internal transcribed spacer (ITS) sequences**. The distances within the tree were constructed using the neighbor-joining method based on Kimura's correction with the CLUSTAL W computer program. The horizontal lengths are proportional to the evolutionary distances. The numbers above or below the internal nodes indicate bootstrap values (1000 replicates)of > 50%.

The 18S rRNA gene and ITS sequences obtained for strain HSO-3-1 were submitted to the National Center for Biotechnology Information (NCBI) under accession numbers JF930340 and JF930341, respectively. The amplified 18S rRNA gene sequence was found to have > 99% identity with that of previously sequenced *Mychonastes *sp. and *Pseudodictyosphaerium *sp. strains in the NCBI database. The 18S rRNA gene analyses of 26 strains belonging to the genus *Mychonastes *clade allowed the recognition of lineages attributable to 10 different species (Figure 1). On the 18S rRNA gene tree, HSO-3-1 was found to be most similar to the *M. afe*r strain CCAP 260/6 (GQ477044.1). To confirm the results of the 18S rRNA gene analyses, the ITS1-5.8S-ITS2 sequence of 26 strains belonging to the genus *Mychonastes *clade was studied, and allowed the recognition of lineages attributable to eight different species (Figure 2). Strain HSO-3-1 was again found to have a close relationship with *M. afe*r strain CCAP 260/6 (GQ477044.1) using this analysis.

### Morphology and cellular structure

**Figure 3:**
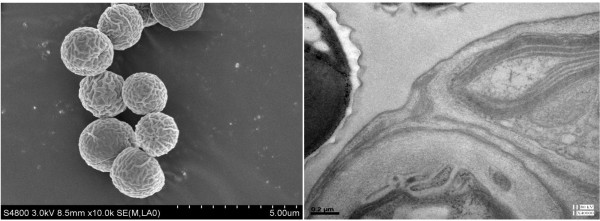
**Microscopy and morphological appearance**. (Left) Scanning electron micrograph and (right) cell-wall morphology of *Mychonastes afer *strain HSO-3-1.

**Figure 4:**
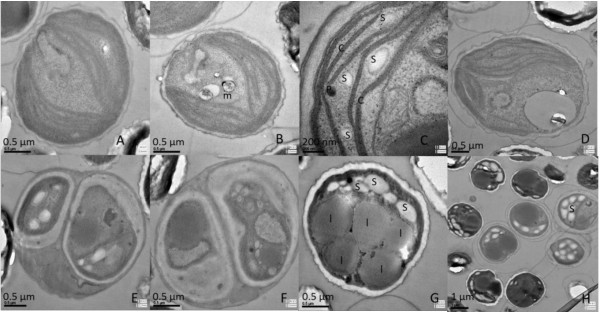
**Organelle morphology of *Mychonastes afer *HSO-3-1**. **(A-D) **Chloroplast (c), starch grains (s), lastoglobule (p) andmitochondrion (m). **(E-F) **Autospores in parent cells. **(G-H) **Lipid droplets (l) in *M. afer *HSO-3-1.

Cells of *M. afer *HSO-3-1 are unicellular, solitary and spheroidal, and the cell size is very homogeneous, varying from 2 to 3 μm in diameter. The cell wall is smooth, and has a layer of along the central axis when examined under scanning electron microscopy (Figure 3, left). The cell wall consists of a double layer (Figure 3, right). The outer layer, which is 50 to 200 nm wide, was found to be a trilaminar sheath, consistent with a previous report [17]. Each cell has a single chloroplast that is crescent-shaped and anchorage-dependent (Figure 4A-D). The chloroplast consists of thylakoid lamellae arranged in six almost parallel rows with pyrenoids (Figure 4A-D). Reproduction takes place by the formation of two autospores (Figure 4E-F). Lipid bodies are often present in the cytoplast after cultivation for a significant period (Figure 4G-H).

Cells of strain HSO-3-1 are single (seldom found in colonies) and planktonic. The adult cells are spherical, 2 to 5 μm in diameter, with a thin mucilaginous envelope. The shape of HSO-3-1 was found to be similar to that reported for *M. afer *[18].

Morphological features of strain HSO-3-1, including the shape of the cells, the composition of the cell wall, and the shape and composition of the chloroplast, are identical to those reported for the genus *Mychonastes *[18] and the species *Mychonastes homosphaera *[19]. However, the autospore formation of HSO-3-1 involves binary fission, and is different to that of *M. h20omosphaera*. The size of the cells is more homogeneous, and the cell wall is thicker than *M. homosphaera*. In contrast to the genus *Pseudodictyosphaerium*, which is characterized as having a parietal, cup-shaped or girdle-shaped chloroplast without pyrenoids, the chloroplast of HSO-3-1 consists of thylakoid lamellae with pyrenoids. Based on these molecular and morphology characteristics, strain HSO-3-1 was therefore identified as *M. afer*.

### Fatty acid identification

The doubling time of *M. afer *HSO-3-1 was found to be about 12.9 hours during the exponential growth phase. After cultivation for 13 days, the microalgae were harvested and lyophilized. The lipids were extracted using a chloroform-methanol-water solvent system, and then converted into their methyl esters. The total lipid content of the strain was about 53.9% dry cell weight (DCW) in BG-11 medium (1.5 g/L NaNO_3_). The FA composition of *M. afer *HSO-3-1 was determined using gas chromatography-mass spectrometry (GC-MS), and 17 individual FAMEs were detected (Table 1).

**Table 1:** Fatty acid composition of *Mychonastes afer *HSO-3-1.

Lipid number	FAME^1^	Relative content (%)
1	C14:0	0.15
2	C16:0	13.24
3	C16:1	5.27
4	C16:2	0.89
5	C16:3	0.46
6	C16:4	0.86
7	C18:0	4.92
8	C18:1	57.55
9	C18:2	4.97
10	C18:3	4.23
11	C18:4	0.53
12	C20:0	0.23
13	C22:0	0.18
14	C22:1	0.64
15	C24:0	0.49
16	C24:1	3.78
17	C26:0	1.59

^1^FAME, fatty acid methyl ester.

The major FA component was C18:1, which accounted for more than half of the total FAME content in *M. afer *HSO-3-1. Biodiesel quality is determined by FA composition [17]. It has been suggested that high-quality biodiesel is usually composed of 18 carbon acids, including oleic acid methyl ester and octadecenoic acid methyl ester [20]. The major FAMEs contained in *M. afer *HSO-3-1 were shown to be hexadecanoic acid methyl ester, octadecenoic acid methyl ester and octadecenoic acid methyl ester, which accounted for over 57.6% of the total lipid content.

The second major FA was C16:0. The analysis also revealed the presence of C24:1 (nervonic acid, 14.7 mg/g of total lipid, 3.8% of total FAs), which has been shown to have biomedical applications [21,22]. The production of nervonic acid by *M. afer *HSO-3-1 increases the value of this microalgal strain for biodiesel production. Nervonic acid is a long-chain monounsaturated FA, which is found in the seed oils of *Lunaria annua *(honesty), *Borago officinalis *(borage), hemp, *Acer truncatum *(Purpleblow maple) and *Tropaeolum speciosum *(Flame flower). Only *Lunaria annua *L. has been studied and grown sparingly for future development as a niche crop [21,22]. Therefore, the discovery of C24:1 in microalgae expands the variety of natural sources of C24:1, and thus improves the potential value of this microalgal strain in biodiesel production, as it can also be used for pharmaceutical production [17].

### Effect of NaNO_3 _concentration on lipid accumulation

**Figure 5:**
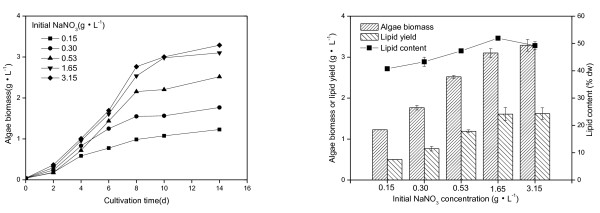
**Characteristics of *Mychonastes afer *HSO-3-1 in BG-11 medium**. (Left) Growth (solid line), algae biomass, lipid yield, and (right) lipid content of *Mychonastes afer *HSO-3-1 in BG-11 medium with different concentrations of NaNO_3_.

The effect of nitrogen concentration on the growth and lipid accumulation of *M. afer *HSO-3-1 was investigated. HSO-3-1 was grown at different NaNO_3 _concentrations. When the concentration of NaNO_3 _was in the range 0.15 to 3.15 g/l, the lipid content of the algae was between 41.7 and 51.9% DCW after cultivation for 14 days, with the highest biomass (3.29 g/l) and lipid yield (1.62 g/l) achieved when the concentration of NaNO_3 _was 3.15 g/l (Figure 5). The highest lipid content (51.9% DCW) was achieved at a NaNO_3 _concentration of 1.65 g/l.

The FA composition of *M. afer *HSO-3-1 cultivated at different nitrogen concentrations was also analyzed (Table 2). The major components were octadecenoic acid and hexadecanoic acid. Octadecenoic acid content ranged from 44.7% to 35.8% and decreased at higher concentrations of NaNO_3_. Hexadecanoic acid increased from 17.8% to 22.6% with increased NaNO_3 _concentration. Hexadecadienoic acid and octadecadienoic acid both increased with increased NaNO_3 _concentrations, whereas other unsaturated FAs displayed the opposite trend. The ultralong FAs obtained from HSO-3-1 were lignoceric acid, nervonic acid and cerotinic acid. Ultralong-chain FA content decreased at increased NaNO_3 _concentrations. Varying the concentration of NaNO_3 _changed the lipid content of the algae and the composition of the major components of FAs.

**Table 2:** Effects of different NaNO_3 _concentration on fatty acid compositions of *Mychonastes afer *HSO-3-1.

Fatty acids	Relative content (%)				
	
	0.15 g/L NaNO_3_	0.30 g/L NaNO_3_	0.53 g/L NaNO_3_	1.65 g/L NaNO_3_	3.15 g/L NaNO_3_
C14:0	0.23	0.22	0.21	0.21	0.20
C16:0	17.81	18.63	17.14	20.34	22.61
C16:1	7.44	7.56	6.82	5.89	5.90
C16:2	1.51	1.66	1.90	1.95	2.65
C16:3	1.78	1.92	2.32	1.42	1.36
C16:4	4.68	4.68	5.43	3.85	4.38
C18:0	3.66	3.62	3.75	4.19	2.95
C18:1	44.71	43.46	42.69	41.58	35.82
C18:2	4.89	5.38	6.75	10.61	13.48
C18:3	8.57	8.01	8.07	6.32	5.91
C18:4	0.99	1.40	1.81	1.89	2.04
C22:1	0.32	0.35	0.28	0.13	0.24
C24:0	0.33	0.28	0.07	ND^1^	ND
C24:1	2.06	2.05	1.89	1.95	1.93
C26:0	1.03	0.96	0.87	0.37	0.53

^1^ND, not detected.

**Figure 6:**
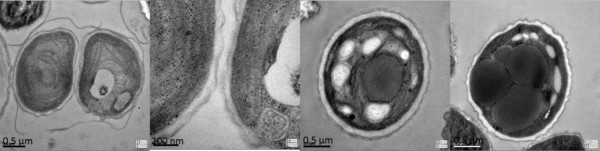
**Cell-wall thickness of *Mychonastes afer *HSO-3-1 under conditions of different lipid content**. Growth of algae **(A, B) **in the early stages with no lipid in the cell; **(C, D) **in the late stages, showing various lipid bodies in the cell.

The total content of lipids in microalgae may vary from about 1 to 85% of dry weight [21,23]. Hu *et al*. summarized the lipid contents of oleaginous green algae reported in the literature, and found that oleaginous green algae had an average total lipid content of 25.5% DCW [17,24]. Nitrogen limitation/depletion has been shown to affect both lipid composition and lipid content of many algae [25]. The promotion of FA synthesis seen during the decay phase was probably due to triglyceride-FA accumulation [15,26]. Rodolfi reported that the lipid content of *Nannochloropsis *sp. increased from 32% to 60% when it was transferred from 'nitrogen-sufficient' to 'nitrogen-deficient' culture conditions. Usually microalgae accumulate lipids under nitrogen-limiting conditions, when energy and carbon sources are available [27]. However, limitation or depletion of nitrogen did not seem to induce lipid accumulation in strain HSO-3-1. Interestingly, using transmission electron microscopy (TEM), we found that the cell wall became thicker when the size and number of lipid bodies dramatically increased in *M. afer *HSO-3-1 (Figure 3, Figure 6). It is unclear whether there is any correlation between the cell-wall structure and lipid body formulation. Thus, the metabolism of lipid accumulation in *M. afer *HSO-3-1 under different nutrient conditions should be investigated further. The lipid content of the new species, *M. afer *HSO-3-1 was up to 51.9% DCW when the alga was grown in BG-11 medium, indicating that the strain could be used as a suitable feedstock for biodiesel production.

## Conclusions

The naturally occurring microalgal strain *M. afer *HSO-3-1 was found to be a good candidate for biodiesel production, because of its high lipid content, yield, and content of suitable FAs and the valuable co-product nervonic acid. The effect of nitrogen concentration on the lipid yield of *M. afer *HSO-3-1 was investigated in photoautotrophic culture systems. Strain HSO-3-1 produced the highest biomass (3.29 g/l) and lipid yield (1.62 g/l) when the sodium nitrate concentration was 3.0 g/l in BG-11 medium. Further research should study the effect of other elements such as phosphorus and iron on biomass, lipid yield and the cell-wall structure, and also investigate how the nervonic acid content of this strain might be improved.

## Methods

### Organism and culture conditions

Strain HSO-3-1 was isolated from wastewater samples collected from Jinan, Shandong province, and stored in our laboratory. The strain was grown in BG-11 medium [28], and maintained at 25 ± 1°C under continuous illumination provided by daylight fluorescent tubes at 70 to 80 μmol photosynthetically active radiation (PAR) photons per m^2 ^per second.

### Electron microscopy

For TEM, cells were fixed with 1% glutaric dialdehyde for 1 hour, separated by centrifugation for 5 minutes at 903×g, then washed three times with phosphate buffer (pH 7.2 to 7.4). The processed materials were fixed with 1% chenic acid for 1 hour and then washed three times with phosphate buffer (pH 7.2 to 7.4). The cells were dehydrated with increasing concentrations of ethanol up to 100% and anhydrous acetone. They were then soaked in an anhydrous acetone:Epon812 resin (7:3) mixture for 5 hours, followed by an epoxy propane:Epon812 resin (3:7) mixture for 6 hours, then Epon812 for 5 hours, after which they were finally embedded in Epon812 resin. Photographs were taken under the microscope (H-7650; Hitachi, Tokyo, Japan). All experiments were carried out at room temperature.

For scanning electron microscopy, the cells fixed with glutaric dialdehyde were embedded using the GTGO (glutaraldehyde, tannic acid, guanidine hydrochloride, osmium tetroxide) technique [29], coated with gold, and examined under a microscope (S-4800; Hitachi, Japan).

### Genomic DNA preparation

Samples (3 ml) of cell cultures were harvested in the mid-late exponential phase, and pelleted by centrifugation. The pelleted cells were suspended in 0.5 ml of Tris-borate-EDTA buffer. The mixture was mixed by vortexing, and the genomic DNA was then extracted (Fast-Prep 24 DNA purification; M.P. Biomedicals, USA) [30]. The purified genomic DNA was used for PCR analysis. The 18S rRNA and ITS region was amplified using universal green algal primers (Table 3), as described previously [31]. The distances within the phylogenetic tree were constructed with the neighbor-joining method based on Kimura's correction, using the CLUSTAL W computer program [32].

**Table 3:** Primers for genomic DNA amplification.

Primer	Sequence 5' → 3'
18SF	CCTGGTTGATCCTGCCAG
18SR	TTGATCCTTCTGCAGGTTCA
ITS4	TCCTCCGCTTATTGATATGC
ITS5	GGAAGTAAAAGTCGTAACAAGG

### Induction of lipid production at different NaNO_3 _concentrations

Microalgae were cultivated in Erlenmeyer flasks to the exponential phase of growth, then transferred to 500 ml glass bubble columns (41 mm in diameter) containing 250 ml of BG-11 medium with different concentrations of NaNO_3 _at a 1:6 (v/v) dilution, to maintain the OD_750 _value at around 0.2. The nitrogen concentration was monitored during growth by measuring OD_220 _and OD_275 _after adding 0.02 mol/l HCl and 1.65 × 10^-4 ^mol/l sulfamic acid according to Feng's method [33,34].

The columns were maintained at 25 ± 1°C, and bubbled with sterile gas composed of air supplemented with 2% (V/V) CO_2_. Continuous artificial illumination at 270 ± 20 μmol PAR photons/m^2^/s^1 ^was provided by daylight fluorescent tubes on one side. All experiments were performed in duplicate.

Growth was estimated by measuring the dry cell weight (DCW). A 5 ml sample was taken every 12 hours and filtered through pre-weighed 0.8 μm microporous filter paper. The filter paper was oven-dried overnight at 105°C. The difference between the final weight and the weight of the paper before filtration was taken as the DCW.

### Lipid extraction

The microalgal cells were harvested after 13 days by centrifugation at 4722×g for 10 min. Cell pellets were lyophilized using a freeze drier (Alpha1-2LD Plus; Martin Christ GmBH, Osterode, Germany). The total lipids contained in the algal cells were extracted with a modified chloroform-methanol-water solvent system [35]. In a 10 ml tube (tube 1), 30to 40 mg dry algal powder, 4 ml chloroform and 2 ml methanol were mixed, and shaken for 10 seconds to disperse the powder. The tube was then incubated for 12 hours at 30°C while shaking at 200 r/min and separated by centrifugation for 10 minutes at 903×g, then 6 ml of the supernatant was collected and transferred to tube 2. Into this, 2 ml methanol and 3.6 ml deionized water were added to give a final chloroform:methanol:water ratio of 10:10:9. The contents were separated by centrifugation at 903×g for 5 minutes, then the chloroform layer was removed, transferred into a pre-weighed tube (tube 3; weight 1) and dried for 30 min under a flow of N_2 _at 60°C. Tube 3 was dried under vacuum (Lantian DZF-6050, Hangzhou, China) for 1.5 hours at 60°C, and then weighed (weight 2). The lipid weight was calculated by subtracting weight 1 from weight 2.

### Fatty acid analysis

Methyl esters were generated from the microalgae lipids by heating them in a 2% H_2_SO_4_-methanol solution at 85°C for 2.5 hours. Analysis of the FAMEs was then performed using GC-MS (7890A-5975C, Agilent Technologies Inc., Wilmington, DE, USA) with a fused column (30 m × 0.25 mm; HP-INNOWAX; Agilent Technologies Inc.). The carrier gas was helium, and 1 μl of the methyl ester sample solution was injected for each analysis. The split ratio was 1:20. The temperature program was as follows: the column temperature was maintained at 50°C for 1 minute, increased to 200°C at a rate of 25°C/min, then increased to 240°C at a rate of 3°C/min, and finally maintained at this temperature for a further 15 minutes. The injector temperature was set at 260°C. The identification of FAs was performed by comparing the mass spectra obtained with Wiley libraries. http://www.wiley.com/WileyCDA/WileyTitle/productCd-1118143949.html

## List of abbreviations

FA: fatty acid; FAME: fatty acid methyl ester; GTGO: glutaraldehyde: tannic acid: guanidine hydrochloride: osmium tetroxide; ITS: internal transcribed spacer.

## Competing interests

The authors declare that they have no competing interests.

## Authors' contributions

CY designed and carried out experiments, analyzed the results and wrote the manuscript. JL and YF carried out the experiments and analyzed the results. XR and GH helped to draft the manuscript. FL designed experiments, analyzed the results and reviewed the manuscript. All authors read and approved the final manuscript.
